# Contextualizing barriers and facilitators to scaling community-engaged research transformation at a historically black medical school

**DOI:** 10.1017/cts.2025.32

**Published:** 2025-03-24

**Authors:** Tabia Henry Akintobi, Rhonda Holliday, LaShawn Hoffman, Latrice Rollins, Yvette Daniels, Howard Grant, Melissa Kottke

**Affiliations:** 1 Morehouse School of Medicine/Prevention Research Center, Atlanta, USA; 2 Hoffman and Associates, Atlanta, GA, USA; 3 Georgia Department of Public Health, Atlanta, GA, USA; 4 Fulton Atlanta Community Action Authority, Atlanta, GA, USA; 5 Jane Fonda Center, Emory University, Atlanta, GA, USA

**Keywords:** community-based participatory research, community-engaged research, institutional determinants, health equity, translational research

## Abstract

**Introduction:**

Morehouse School of Medicine (MSM) embodies an applied definition of community engagement advanced over four decades. The increased demand for community collaboration requires attention to the *institutional contexts* supporting community-engaged research. MSM partnered with the University of New Mexico Center for Participatory Research for the Engage for Equity (E2) PLUS Project to assess, ideate, and consider existing and recommended institutional supports for community-engaged research.

**Methods:**

MSM assembled a community-campus Champion Team. The team coordinated virtual workshops with 18 community and academic research partners, facilitated four interviews of executive leaders and two focus groups (researchers/research staff and patients/community members, respectively) moderated by UNM-CPR. Analyses of the transcripts were conducted using an inductive and deductive process. Once the themes were identified, the qualitative summaries were shared with the Champion Team to verify and discuss implications for action and institutional improvements.

**Results:**

Institutional strengths and opportunities for systemic change were aligned with equity indicators (power and control, decision-making, and influence) and contextual factors (history, trust, and relationship building) of The continuum of community engagement in research. Institutional advances include community-engagement added as the fourth pillar of the institution’s strategic plan. Action strategies include 1) development a research navigation system to address community-campus research partnership administrative challenges and 2) an academy to build the capacities of community/patient partners to *independently* acquire, manage, and sustain grants and negotiate equity in dissemination of research.

**Conclusions:**

MSM has leveraged E2 PLUS to identify systems improvements necessary to ensure that community/patient-centered research and partnerships are amplified and sustained.

## Introduction

The differential distribution of the social determinants of health (SDOH) is grounded in racism and other historic and currently sustained socially and politically constructed conditions that systematically marginalize and exclude groups defined by a complex intersection of individual and contextual factors. They work together to contribute to our nation’s health disparities.[1–4] Community-engaged research (CEnR) and community-based participatory research (CBPR) have been increasingly utilized in efforts to upend the SDOH that serve as barriers to optimal health and healthcare, trust in research, and its effective translational toward advancing health equity.[5–9]

Historically Black Colleges and Universities (HBCUs) are uniquely positioned to address health disparities and inequities through community-engaged research due to their proximity to communities that are disproportionately affected by chronic diseases, poverty, limited access healthcare, their social missions and attention to addressing SDOH through service, community engagement, and the preparation of health professionals poised to advance primary care and prevention.[10–11] Several researchers have explored and advocated for the role of HBCUs in promoting CEnR.[12–14]

Embedded in the Morehouse School of Medicine (MSM) mission is the improvement of the overall health and well-being of individuals and communities; increasing the diversity of the medical, scientific, and public health workforce; and training future health learners and leaders. Its vision is to *lead the creation and advancement of health equity to achieve health justice*. This is accomplished through the execution of programs in education, research, and service in collaboration with partners that address both standard health outcomes and, as importantly, root causes of health inequities (systems, conditions and contexts) reflecting the SDOH. The MSM Office of Community Engagement (OCE) facilitates community-centered communication, coordination and collaboration for education, clinical, research, and service initiatives. This foundation was further strengthened by coining the T^X TM^ translational research scholarship tenets symbolizing a method and scientific philosophy promoting the convergence of interdisciplinary approaches and scientists *with* community/patient integration to stimulate exponential advances with evidence or implications for transforming the health of prioritized communities or patients.[15] The tenets were further demonstrated by exemplars through the Multidisciplinary Translational Teams.[16]

**Figure 1.**
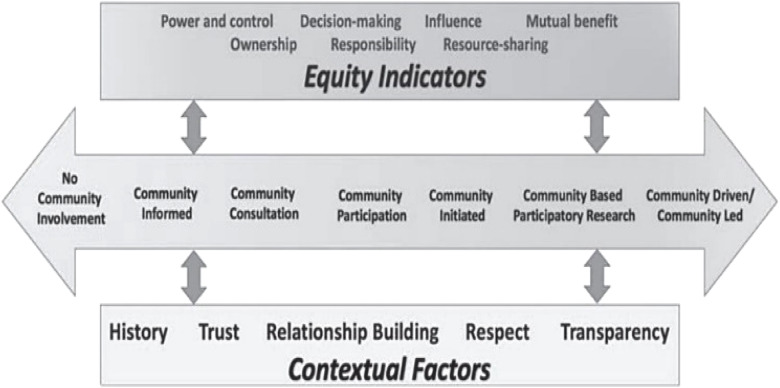
The continuum of community engagement in research[17].

The MSM Prevention Research Center (MSM PRC), funded by the Centers for Disease Control and Prevention (CDC) since 1998, is the designated Center for CBPR and CEnR within MSM. The center regularly collaborates with other MSM institutes and centers including the Cardiovascular Research Institute, the National Center for Primary Care, and the Satcher Health Leadership Institute, to advance CEnR, translation and dissemination with community leaders and partners. Its leadership or collaboration on several federal and privately funded initiatives to model and build capacities for community-driven research and infrastructures include, but are not limited to, the Clinical and Translational Science Institutes, the Centers for Diabetes Translational Research (including RADxUP), the Center for Translational Research in Health Disparities, the Community Engaged Alliance Against COVID-19 Disparities Network, and the National COVID-19 Resiliency Network demonstrating commitment to building and sustaining community-engaged partnerships. The conceptual model in Fig. 1 delineates both the equity indicators and contextual factors that serve as both partner and institutional determinants of successful CEnR and will be referenced throughout this report.

## Equity indicators

### Power and control

The MSM PRC is ***governed*** by its Community Coalition Board; established in 1998, it convenes every two months. The CCB is not just an advisory board but is structured as a ***policy-making*** board. The 19-member CCB has systematically led (following a community-majority vote towards approval) conceptualization, planning, implementation, and evaluation of funded and unfunded public health research, evaluation, and practice strategies aligned with its community values, evaluation criteria, and guiding principles.

### Influence

The board comprises three types of members that reflect a disease agnostic approach to CEnR governance. *Community residents* reflecting those at disproportionate risk and response are prioritized as priority ranking members. CCB Bylaws stipulate that neighborhood representatives will always be in the majority on the Board and that the Board Chair, Vice-Chair, and Secretary will always be non-faculty/staff neighborhood representatives. Hence, in any vote that pits the neighborhoods against the academics and public health professionals, the neighborhoods would have the most votes (however, this has never occurred). *Academic institutions* support and ensure research and evaluation rigor, evidence-based translational and implementation science and clinical care linkages central to immediate response to community priorities-through and beyond research projects. *Public health, Policy, Systems, Environmental Change Agencies* that include but are not limited to state and local health departments, and social services (housing, economic development, food access, among others) allow for community or patient groups to be heard directly towards SDOH linkages and action for advocacy and policy changes.

### Decision-making

The CCB consists of four standing committees including the Communications and Technology Committee, Data Monitoring and Evaluation Committee, The Project Review Committee and Executive Committee (composed of CCB and MSM PRC leadership). They are led or co-led by Board members to ensure academic institution accountability to community values and priorities and community-centered leadership in decisions related to all MSM PRC functions. The CCB integral leadership in directing the center’s work in research, communication, training, and evaluation are detailed in Table 1.

**Table 1.** Community Coalition Board (CCB) level of involvement in Morehouse School of Medicine Prevention Research Center (PRC)

Key element	CCB level of involvement
Research	Establishes core research agenda; establishes research priorities; reviews all projects and protocols; may modify or reject a project/protocol; community-engaged research committees (deployed to lead specific research projects from conception dissemination); leads community-engaged research.
Communication	Serves as subject matter expert on topic development for presentations, workshops, webinars, media and web/social media content, and/or messaging; Co-authors manuscripts and written documents, e.g., journal articles, bulletins, fact sheets; presents findings and promotes health at scientific and community presentations, on radio, TV and through other media, and social media outlets; disseminates research findings and health materials in communities served by PRC and serves on Communications and Technology Committee (established in 2010 and co-chaired by CCB member; 20 newsletters; 80 radio shows).
Training	Supports recruitment of community health workers personnel; co-leads students and community courses and trainings on designated PRC research priority topics established by CCB.
Evaluation	Co-leads development of surveys for appropriate data collection; participating in and leading data collection; presenting evaluation findings in community and academic setting; providing input as CCB member in the PRC’s collection of data from the CCB and assisting in programmatic decision-making based on evaluation findings that impact the PRC and/or the CCB.

The new, exponential demand for MSM patient and community leadership or collaboration in research initiatives addressing root causes of health and healthcare disparities including social injustice, racism, among others has required new attention to the *institutional systems and practices* central to enabling and sustaining community centered collaboration development and sustainability. To address these issues, the University of New Mexico Center for Participatory Research (UNM CPR) collaborated with MSM PRC to assess, ideate and strategize regarding the institutional support and processes necessary to bolster support to initiate and sustain patient and community-engaged research partnerships.

The MSM PRC was one of four Academic Health Center (AHC) sites for the UNM CPR Patient-Centered Outcomes Research Institute (PCORI) award based on an over three-decade history of community-campus research collaboration.[17] This collaboration built upon MSM PRC’s s role as a contributor to and beneficiary of the UNM CPR Engage for Equity (E2) network which identified CBPR promising practices[18] and tested the resulting toolkit with over 200 funded research partnerships.[19–21] The E2 intervention was developed for and tested at the community-campus partnership level. The E2 PLUS Project was designed to assess, ideate and strategize regarding the *institutional* supports and processes determinants central to initiating and sustaining patient and community-engaged research partnerships. The purpose of this manuscript is to detail qualitative perspectives garnered from community/patient, academic research, and executive leaders on existing institutional contexts and those recommended to develop or strengthen supportive policies and practices to support CEnR and CBPR.

## Materials and methods

The MSM PRC assembled a seven-member community-campus Champion Team to support leadership, reflection and engagement with the UNM CPR and other AHC sites. Five members were strategically selected CCB members reflecting each member type detailed earlier. Two members were CBPR/CEnR researchers with a history of equity-centered community research partnership.

In the first year of the E2PLUS intervention, the Champion Team facilitated meetings to plan community, academic researcher and stakeholder engagement strategies. Virtual AHC workshops were conducted in October and November 2021 engaging 18 community campus partners, facilitated by UNM CPR. The purpose was to ground teams in reflection on the history, barriers, and determinants of CEnR and supports and MSM. Four interviews of executive leadership (President, Dean, Executive Leadership Team Member, and Strategic Plan Executive) were conducted to garner their perspectives on CEnR promoting policies, practices, and resources as aligned with their vision for health equity (see Supplementary Material 1).

The Champion Team supported the planning and facilitation of two focus groups and three key informant interviews in the second year (see Supplementary Material 2). The two focus groups were comprised of researchers/research staff and patients/community members, respectively (6–8 members per group). The purpose of the groups was to facilitate reflection on perceived barriers and facilitators to CEnR or CBPR. The studies involving human participants were reviewed and approved in collaboration with each AHC by University of New Mexico Health Sciences Center (HRRC: # 21-320). Written informed consent for participation was not required for this study in accordance with national legislation and institutional requirements.

UNM-CPR conducted analyses of the transcripts using an inductive and deductive process based on elements of the CBPR Model (contexts, partnership processes, intervention/research, and health or social justice outcomes) and E2 PLUS framework (collective past, current, and future state reflection and action)[22]. Once the themes were outlined, the qualitative summaries were shared with the MSM Champion Team to verify and discuss implications for action and institutional improvements. Tensions and action strategies were subsequently developed by the Champion Team. The Continuum of Community Engagement in Research, detailed earlier in this report, was the anchoring analytic framework used to analyze and describe results in the section that follows.

## Results

### Virtual AHC workshops

## Contextual factors

### Relationship building and trust

Participants agreed on the need to build upon the position of MSM as an anchoring CEnR lead institution with community and national/global networks central to established and sustained trust. Based on its nearly five-decade history of community engagement, it has exponentially moved to national and global visibility due to both the social and public health awakening catalyzed by the COVID-19 pandemic and the social injustice witnessed in the murders of George Floyd, Ahmaud Arbery among others which highlighted the significance of deeply rooted organizations founded in advancing justice and equity long ago. The institutions’ role was seen as central to building the capacities of other academic institutions seeking to build deep, sustained community and patient-centered research processes and outcomes with demonstrable impact. The establishment of the MSM OCE was seen as an essential investment that should not be episodic, but further leveraged to foster community relationship building and community development initiatives, beyond CEnR, thereby fostering trust in research.

## Community/patient focus groups

### Strengths

#### Research transparency and respect

Community and patient focus group participants cited noteworthy strengths facilitating CEnR in collaboration with MSM. First, regular facilitation of the engagement of community constituents (residents, patients, businesses, social service agencies) addressing or experiencing the SDOH and serving as essential knowledge holders in determining the barriers and facilitators of research that matters was a noted institutional value and action. The long-standing infrastructure of the institution in facilitating a formal process of community review and approval of research, through the MSM PRC CCB, was a cited strength. MSM faculty and staff being visible in the community solely for relationship building and sustainability, was also a community-perceived strength of the institution. Quotes associated with these themes are below:

“Being involved with MSM is the empowerment that they give the community and organizations to be able to make decisions. It’s not just them making decisions, but it’s bringing the community together and allowing us to have input into the process.”

“We had different research projects where we worked with businesses, government agencies, law enforcement, and academic institutions. Morehouse recruits members from each of those entities to the table when developing programs and uses strategies to look at those different areas.”

“I think one of the things with MSM is that they’re willing to listen…. They are being vulnerable enough to open it up to the community experts. I think that’s really key.”

“It’s the communities that have the power to make the decisions when it comes to the research that’s going to be done and the projects that we’re taking on…. It has actually shifted certain research projects. So, there’s a very big influence from the community.”

“They are very supportive with boots on the groundwork, more than our research. They’re helping us conduct the research by actually going out into the community with us.

### Challenges/opportunities

#### Resource sharing (communications, training and capacity building)

Community and patients shared challenges and opportunities that centered on the desire for more engagement in research and focused capacity building. Learning more about the broader scope of research led by MSM, beyond the MSM PRC, is desired given general community trust of MSM. The need for a systematic communication plan towards research results and related best practices being shared back to the community, beyond those who were directly involved, was emphasized. Finally, research training and capacity building with a specific outcome of community and patient groups being better equipped to be equitable CEnR partners was recommended. Implicit bias training was requested to provide tools for organizations to have courageous conversations towards understanding the social determinants of injustice, SDOH and a path towards improved CEnR participation. Community advocacy was emphasized as a reason for more MSM community presence. Quotes associated with these sentiments are below:

“Part of our mandate is to translate this. There is a committee now on translating that I sit on. We recently had a meeting with the chairs of the local community’s NPU (neighborhood planning units) … we are talking about the needs in the communities and what kind of research and projects that we have going on. So, there is some movement in this area, but absolutely I’d like to see it expand and become more institutionalized.”

“I think there’s a lot, like the IRB or some of the connections that are happening that I, as a community member, are just not aware of.”

“There is a lot of research conducted in the communities and I was concerned that they never get to know the outcomes.”

“You have all these researchers and people with a lot of experience. If someone from the community does not have that same experience it can be intimidating.”

“Also getting trained and adding to their programs on implicit bias, even just introducing it to all parties. Even just introducing it, being vulnerable enough to introduce it to all the parties whether they’re the IRB, a community agency, law enforcement, medical providers, any of them.

“I would like to see MSM at the table in the community more, in various ways. Community advocacy for one. “

## Investigator focus groups

### Strengths

Investigators conducting CEnR identified strengths at MSM. They noted the institution valuing their engagement with community partners. They also acknowledged the strength of the MSM PRC CCB in facilitating ongoing community-governed planning and implementation of their research.

### Challenges/opportunities

#### History

Challenges cited, particularly post-pandemic, centered on local and national recruitment of minorities in clinical trials given both historical and pandemic induced trauma reinforcing research mistrust. While CEnR was a well-recognized strength for T3 or T4 researchers (clinician or social behavioral science), there was uncertainty on how to support and expand new models of CEnR translation among basic scientists. Investigators, like community leaders, noted the need for improvement in communicating CEnR activities in centers beyond the MSM PRC. Primary recommendations were to develop forums through which the broader institution learns about CEnR /CBPR as grounds for collaboration, training and capacity building with an emphasis on advocacy training for health policy development.

“Making sure that everyone in the institution is really aware of what the (community engagement) office does and can articulate what it does, from senior leadership all the way down.”

“How do you start these conversations with the people who have been doing community engagement for a while, and how you approach them with a new idea, which is different from the standard, or from their point of view? How do you start that conversation and how do you convince an institution that we need to try new methods in community engagement?”

“I think the challenge we have in working in communities that are not traditionally trusting of some of these medical activities that we are trying to do based on clinical research history, as you know.”

“The more we can collaborate and link our research and partnerships to policy the more we’ll be able to use the lived experience to help people understand why change is important.

## Leader interviews

### Strengths

#### History

Institutional leaders reflected on the strengths of having people aligned with a strategic plan grounded in an audacious vision - *to lead the creation and advancement of health equity to achieve health justice* as a central strength of CEnR at MSM. This commitment, considered to be in the veins, bricks and mortar of the institution, made it a ready responder and collaborator during the COVID-19 pandemic. This resulted in the acquisition of and exponentially increased number of CEnR grants in response to community-identified needs and leveraging their strengths. The establishment of T^X TM^ philosophy and scholarship was also considered an important milestone and identity of the institution. Quotes associated with these sentiments are below:

“We have been in the health disparity space since our existence, it’s part of our DNA. We believe that to some extent Covid has allowed others to see what we have known for the last 40+ years.”

“But at MSM, you know, it is in the fabric of the institution. That’s what we were founded for, and that does matter. And I think from our vision and mission, we are unapologetic about it. I think we put our money where our mouth is.”

“We launched our strategic plan to achieve health equity. It is actionable. It requires that you do something that you have to measure, and it should have an outcome.”

“We coined Translation as Transformation, T^X TM^ The “X” also represents the exponential scale it takes to create health equity.”

### Challenges/opportunities

#### Administrative hurdles and resources

Leaders described challenges poised for actionable solutions. First, administrative strengthening of both the human and fiscal infrastructures was a central theme to acknowledge the disparate organizational contexts of academic institutions and community-based partners. Administrative turnaround times or delays in the processing of community contracts, consultant agreements or payments were noted issues to be addressed. Further, ensuring that not only leaders (Principal Investigators [PIs] or administrative leaders) of CEnR grants, but staff (program managers, community health workers) and community partners be part of strategic planning, policy development and institutional decision making due to their wisdom in brokering, sustaining, and navigating trust and administrative issues of CEnR community partners was emphasized to advance engagement equity. Institutional investments in human capital, beyond extramural grants, central to CEnR facilitation, was seen as a need to ensure that PIs and their staff, due to their increasing demand post-pandemic, do not burn out. Representative quotes are below:

“The one barrier I’m trying to work through, we don’t have human capacity, don’t have the bench needed to really operate in a way that keeps people from becoming burnt out.”

“I do not think that we had demonstrated that we saw the value of everybody. So, it’s been a journey to get people to understand that everybody gets invited to the table.

“It’s just a matter of understanding there are lots of different tables, and stress that importance to the leaders.”

## Discussion

Tensions reflected by themes, across stakeholder types, have centered plans to strengthen existing institutional supports for CEnR and CBPR. Institutional strengths and opportunities for systemic change were aligned with equity indicators (power and control, decision-making, influence) and contextual factors (history, trust, relationship building) of The Continuum of Community Engagement in Research. These factors reflect the values that found the CEnR partnerships and institutional contexts that both must be attended to advance equity-driven processes and outcomes.

While health equity has been explicitly articulated in the MSM vision for ten years, institutional resources (as distinct from extramural grants) to meet the community demand for increased presence and collaboration, beyond research, have been prioritized. MSM has trained, hired and retained hundreds of Community Health Workers (CHWs) as essential members of research, clinical and service teams for over three decades with primary integration through extramural funding[23–24]. Beyond the research enterprise, the institution had built upon the establishment of the first national high school CHW training program[25] certification to establishment of county and statewide CHW apprenticeship, creating pathways from training to job placement and retention.

The MSM OCE has leveraged institutional resources due to increased institutional political will, to hire a director and CHWs who participate in recurring meetings and initiatives designed to identify communities’ priorities and leaders and facilitate partnerships to advance policy, advocacy, and response, following the recent closure of the primary hospital serving the largest number of indigent communities in South Atlanta in 2022. The next year the OCE began partnership with a local library system, at their request, to provide education and capacity building on health literacy and sexual health in response to reports of increased incidence and prevalence of HIV/AIDs infection in a four-county area. The OCE hosts a quarterly Talk, Learn and Collaborate series featuring community and/or academic leaders advancing health equity topics and action strategies. Offered to both a face-to-face and a virtual audience, its reach and audience has broadened potential CEnR opportunities.

Second, while there is unwavering support for CEnR, relatively fewer academic researchers, relative to basic and clinical scientists, represent this science, with a demand that currently far outpaces faculty recruitment and capacities. In light of these challenges, MSM has sought to increase the capacity of current faculty through institutional funding. Institutionally funded T^X TM^ pilot projects funded to collaborating basic, clinical and social behavioral scientists and community/patient groups reflected a small investment of $750K resulting in awardees subsequently acquiring research funding totaling over $75 million, to date. Other multidisciplinary translational teams actualizing the T^X TM^ approach with requisite engagement of community partners and student learners were recently featured elsewhere[16]. The T^X TM^ McNairy Science of Disease Seminar Series through the MSM Office of Sponsored Research Development, is designed to facilitate increased translational research awareness and collaborations.

Third, while the aforementioned strength of MSM PRC Community Coalition Board is decades long, there is a need to infuse the community engagement “value proposition” across the institution and beyond, to avoid staff and faculty burn out through increasing the capacities of other faculty to do CEnR. Once an approved project is funded, Community Engaged Research Committees (CERCs) are assigned to guide the project, post-award, and support research translation and dissemination efforts. Composed of a small group (three to five) of board members and other identified leaders, they bring expertise and networks that strengthen specific research studies or relationships with priority populations. This model has been adapted for the Georgia Clinical and Translational Science Alliance (Georgia CTSA), an inter-institutional research magnet that concentrates basic, translational, and clinical research investigators, community clinicians, professional societies, and industry collaborators in dynamic clinical and translational research projects. The Georgia CTSA Community Engagement Program maintains *a web-based* 3-tiered *service system* to streamline CEnR requests, submissions, and responses[26]. making the process accessible to investigators statewide with community representatives compensated for general membership and committee service. More recently, an advisory board of community members was integrated by the MSM PRC co-led Georgia Center for Diabetes Translational Research. Members from agencies such as the Atlanta Area Health Education Center, The Georgia Rural Health Association, faith-based organizations, and community associations engage with pilot project applicants from Georgia Institute of Technology, Emory University, and MSM both before application submission and after projects are funded to ensure attention to validity of community engagement methodologies and health equity implications of research.

The explicit impact of the E2 Plus Project evaluation process is reflected by the concurrent timing of the elevation in positioning of community engagement in the institution’s strategic plan. First, community-engagement was added as the fourth pillar of the institution, alongside education, clinical care, and research is a nationally unique positioning in academic health centers, making its position explicitly represented as an institution priority. MSM PRC co-leadership of the committee, comprised of faculty and staff, institution-wide, to develop community engagement pillar goals and objectives will support integration of the issues and priorities identified through the E2 PLUS Project process. Action strategies prioritized for development include a community/patient research navigation system will be established for partners entering into formal agreements with MSM to address institutional administrative challenges cited here and nationally recognized in community-campus research partnerships[27]. Second, centralized strategies to promote new and established programs to improve the capacities of community/patient partners to *independently* acquire, manage, and sustain grants will move existing models of grant writing support to emerging capacities to ensuring fiscal buoyancy and equity in scholarly dissemination related to CEnR partnerships, processes, and outcomes. The results of the MSM E2 Plus evaluation will be used to initiate and sustain systems change central to ensuring that identified challenges and opportunities result in the changes necessary to realizing (and not solely positioning) equity in the community engagement pillar relative to the others central to the institution’s mission.

## Conclusion

MSM has leveraged the E2PLUS partnership to strategically identify systems improvements necessary to ensure that community/patient-centered research and partnerships, institution-wide, are amplified and sustained. A community-centered translational research system is accomplished through the execution of programs in education, research, clinical care, and service in collaboration with partners that address health and health care outcomes and, as importantly, root causes of health inequities (systems, conditions, and contexts) reflecting the SDOH. Those who embody these values and practice will engage in early and ongoing assessments with communities that deepen understanding of assets, needs, history, and power relations. This community knowledge and wisdom must advance institutional action that also attends to expressed and assessed needs for training and capacity building support for all partners. The work of both individual and institutional systems that embody and act in ways informed by these values will result in community-led data, metrics systems, and networks to inform and advance CEnR, practice and clinical models that strengthen the capacity of all partners to independently and collectively plan, implement, and sustain interventions that eradicate administrative barriers along with structural determinants of poorer health to advance health equity, for all.

## Supporting information

Akintobi et al. supplementary material 1 Akintobi et al. supplementary material

Akintobi et al. supplementary material 2 Akintobi et al. supplementary material

## Supplementary material

The supplementary material for this article can be found at https://doi.org/10.1017/cts.2025.32.
