# Supplementary material for: Contextualizing barriers and facilitators to scaling community-engaged research transformation at a historically black medical school
Source: J Clin Transl Sci. 2025 Mar 24;9(1):e57. doi: 10.1017/cts.2025.32 (PMC11975764; doi:10.1017/cts.2025.32)
Supplement: Akintobi et al. supplementary material 1 — Akintobi et al. supplementary material [file S2059866125000329sup001.docx]

E2 PLUS Focus Group Discussion Guide

# **Section A: Introduction**

## *A1. Introduction to the study and team members*

Welcome and thank you so much for taking the time to participate in this focus group.

In this focus group, we are hoping to gain a better understanding of the context of your institution and what it means for the institution to engage in health-equity oriented, patient and community research.

My name is [your name] and I will be your moderator today. Joining me is/are [insert other team member names], member(s) of our research team.

## *A2. Focus Groups for research*

Focus groups are organized by researchers so that they can learn more about the experiences, opinions, attitudes, and beliefs of a specific group of people about a designated topic. The information obtained from focus groups is then used to guide future action on that topic.

A focus group is not:

· A debate

· Group therapy

· A conflict resolution session

· A problem-solving session

· An opportunity to collaborate

· A promotional opportunity

· An educational session

## *A3. Expectations from research participants*

- In this study your role is to:
  - Represent yourself and respond freely and honestly.
  - Not worry about right or wrong answers because you are the expert in YOUR opinions and experiences.
  - Disagree with others’ opinions but please remain respectful of other participants at all times. We are here to share experiences and perspectives, to listen and to learn.

- In this study, the team members have various roles:
  - The moderator’s role is to facilitate the group and guide the discussion. They will present questions to participants.
  - The moderator is not here to give information or provide opinions. It is YOUR thoughts and opinions that matter in this session.
  - The notetaker’s role is to write down key points from the discussion. They may jump in with their own questions toward the end of the focus group.

## *A4. Confidentiality*

- To maintain confidentiality, we ask that you **do not** share what other people say in this focus group.
- To accurately interpret and analyze the data from this group discussion, we will be recording the audio of this focus group.
  - These recordings will then be transcribed and deleted as soon as transcription has taken place. There will be no identifying information connected to the transcripts
  - The final report may contain some individual quotations from the focus groups, however specific names will not be included.
- Only authorized research team members will have access to the data from this group discussion. The transcripts will be stored on a password protected computer and in a locked cabinet in a secured office at UNM.
- As a reminder, you may discontinue your participation in this focus group at any time for any reason.

## *A5. Focus group logistics*

***Duration of focus group:***

- This focus group will last approximately ___
- Please feel free to excuse yourself if you need a restroom break, or if the discussion is making you uncomfortable.

***Ground rules for participation:***

- Give freely of your thoughts, feelings, opinions, and experiences and give everyone some space to express themselves.
- Speak only for yourself and let others do the same
- Respect and appreciate the other’s points of view.
- Do not use hate speech of any kind and this includes but is not limited to racial, homophobic, or transphobic slurs. You will be asked to leave if you use this type of speech.
- Try to confine your comments to the topic at hand.
- Protect the confidentiality of one another by not sharing other’s responses outside of this group.

# **Section B: Discussion Questions**

# Investigator Focus Group Questions

INTRO QUESTIONS: Ask people to talk briefly about the types of CBPR/community or patient engaged research you have been involved in or led at this institution. (Name of your project (s)/ how long been involved / and which kind of partners).

STATUS of CBPR and REPUTATION questions:

Thinking about people who have high status reputations in your institution, how is that reputation gained?

How do you perceive the institution values CBPR/patient-engaged research? How do they show this value? Or how do they not?

INSTITUTIONAL CONTEXT:

Can you describe the entities, centers, or people in the institution that have played a key role in advancing your CBPR/patient engaged research?

In your opinion, what are the key barriers to operationalizing equity-based patient and Community Engaged Research in your institution?

What changes would you like to see happen in the next year to strengthen equity-based patient and community engagement as a sustained committed effort?

What are the policies and practices that have helped you start and sustain your CBPR/patient engaged research?

Some of us only do CBPR research and for others, multiple kinds of research, for you when you do CBPR research, what are other additional supports you need to execute your research well?

What are the policies, practices, or entities that have been barriers or made it more difficult to do your work?

What decisions are being made about research that you find detrimental?

COMMUNITY CONTEXT:

Let’s talk about your knowledge of community involvement here, does ___ have an institutional community advisory board or at what level, for which centers?

From your experience, what do they do?  What decisions do they make?

Would the CBPR /health equity research look different if communities were driving it?  If so, how?

PERSPECTIVE OF CHANGE: These days there is a lot of emphasis on institutional transformation towards greater equity. How do you envision institutional transformation here?

Are there key initiatives that you are aware of or are participating in?

What changes, if any, do you see are needed in the institution to strengthen community engagement as a sustained committed effort?

We have covered a lot of material today and we are grateful for your time. As a reminder your name will not be included in the transcript and our intent is to better grasp how leaders like yourself prioritize academic health research, view institutional transformation, and how they are implementing CBPR/CeNR mandates.

# Community/Patient Focus Group Questions

INTRO QUESTIONS: Ask people to talk briefly about the types of CBPR/community or patient engaged research you have been involved in as a community member or as a CAB member or a patient advocate? (Name of project/ your role)

COMMUNITY CONTEXT:

From your perspective, how do you think ____ values CBPR/patient-engaged research? How is this shown? Do you think CBPR, and patient-engaged research is valued at all levels of the institution?

Let’s talk about your knowledge of community involvement at ____ Do they have institutional community advisory boards or at what level do these CABS exist?

From your experience, what do they do? What decisions do they make?

As you all know that power comes in many ways, for example, who speaks the most at a meeting, or whether you feel you can even state your ideas, or whether decisions really happen outside the meeting?  Have you experienced these power dynamics? Can you give examples?

Would the CBPR /health equity research look different if communities were driving it? If so, how?

INSTITUTIONAL CONTEXT:

What are the policies and practices that have helped start and sustain CBPR/patient engaged research?

Some of us only do CBPR research and for others, multiple kinds of research, for you when you do CBPR research, what other additional supports do you need to execute your research well?

What are the policies, practices, or entities that have been barriers or made it more difficult to do your work?

What decisions are being made about research that you find detrimental?

In your opinion, what are the key barriers to operationalizing equity-based patient and Community Engaged Research in your institution ?

What changes would you like to see happen in the next year to strengthen equity-based patient and community engagement as a sustained committed effort?

PERSPECTIVE OF CHANGE: Are there any institution initiatives towards advancing equity that you are aware of or are participating in?

What changes, if any, do you see are needed in the institution to strengthen community engagement as a sustained committed effort?

We have covered a lot of material today and we are grateful for your time. As a reminder your name will not be included in the transcript and our intent is to better grasp how leaders like yourself prioritize academic health research, view institutional transformation, and how they are implementing CBPR/CeNR mandates.
