# Supplementary material for: Contextualizing barriers and facilitators to scaling community-engaged research transformation at a historically black medical school
Source: J Clin Transl Sci. 2025 Mar 24;9(1):e57. doi: 10.1017/cts.2025.32 (PMC11975764; doi:10.1017/cts.2025.32)
Supplement: Akintobi et al. supplementary material 2 — Akintobi et al. supplementary material [file S2059866125000329sup002.docx]

E2 PLUS Leadership Interview Guide

# **Section A: Introduction**

## *A1. Introduction to the study and team members*

Welcome and thank you so much for taking the time to participate in this interview.

In this interview, we are hoping to gain a better understanding of the context of your institution and what it means for the institution to engage in health-equity oriented, patient and community research.

My name is [your name] and I will be your interviewer today. Joining me is/are [insert other team member names], member(s) of our research team.

## *A2. Expectations from research participants*

- In this study your role is to:
  - Represent yourself and respond freely and honestly.
  - Not worry about right or wrong answers because you are the expert in YOUR opinions and experiences.

- In this study, the team members have various roles:
  - The interviewer's role is to ask questions
  - The interviewer is not here to give information or provide opinions. It is YOUR thoughts and opinions that matter in this session.
  - The notetaker’s role is to write down key points from the interview. They may jump in with their own questions toward the end of the interview.

## *A3. Confidentiality*

- To accurately interpret and analyze the data from this interview, we will be recording the audio of this interview.
  - This recording will then be transcribed and deleted as soon as transcription has taken place. There will be no identifying information connected to the transcripts
  - The final report may contain some individual quotations from the focus groups, however specific names will not be included.
- Only authorized research team members will have access to the data from this group discussion. The transcripts will be stored on a password protected computer and in a locked cabinet in a secured office at UNM.
- As a reminder, you may discontinue your participation in this interview at any time for any reason.

## *A4. Focus group logistics*

***Duration of interview:***

This interview will last approximately 1 hour.

# **Section B: Interview Questions**

**Questions for Top Leaders**

*An academic health center encompasses all the health-related components of universities, including their health professional education, patient care operations, and research enterprise. Leaders include CEOs, Chancellors, and Executive Vice Presidents.*

Thank you for agreeing to participate in this interview.

To begin, we would like to hear a little bit about you. How long have you been in your current role? What is your official title? How long have you been at (insert name here)? Tell me a little bit about your administrative career. What are your research or clinical interests?

**Basic Organizational Structure**

Thank you for sharing some details about yourself. I’d like to turn to some basic questions about the overall structure and governance of your School of Medicine/Consortium). In general, we define governance as the structures and processes that guide your research, clinical, and policy outputs as an organization.

What entities and positions play a key role in advancing your research mission and vision? Whom do you rely on to implement the overall research mission and vision?

Thank you for sharing some about your organizational structure. Moving beyond institutional arrangements, let’s talk a little more about the processes you use to make decisions and coordinate your institution.

PROBE: (for horizontal decisions) How are decisions made when there is conflict between collective decision-making and the strategic direction, budget, funder interests, or other institutional interests within the School of Medicine/Consortium?

Relatedly, and as I’m sure you are aware, leaders like yourself adopt different styles and ways of governing their organizations. Some leaders prefer to take a proactive role and centralize power in their office, others prefer to delegate authority, some prefer decentralized decision making, while others prefer to take each situation into account before delegating, commanding, or sharing decisions.

Thinking about these styles, how would you describe your overall leadership style?

**Aspirational**

We understand clinical care drives much of the agenda of the medical school/Consortium, with research and education also important in terms of your responsibilities.  Knowing this, thinking aspirationally, if you had complete control of your agenda, where would you focus your efforts? (Try to ascertain whom they feel most accountable to and what the institution's priorities are.)

PROBE:  Tell me more. What is preventing you from focusing on this agenda?

**Perceptions of status, power, and resources**

Given the nature of our project, we would like to spend some time focusing on your academic health centers’ research infrastructure and direction. In your view, what kind of research is your institution most known for? (signature programs) What are some of your key research initiatives?   From your perspective, what are some of your newer and more emerging areas? After pointing out your key strengths, what are areas for improvement?

It would be helpful to understand your School of Medicine/Consortium supports faculty research.  What initiatives /strategies do you have that support faculty? How do you support different kinds of research?

PROBE: What are your areas for improvement?

As you know, a common metric of research capacity is your current level of research funding. Who are your major grant funders? What does your research endowment look like?

**Institutional Transformation/Innovation**

These days there is a lot of emphasis on institutional transformation or innovation in health science. How do you envision institutional transformation or innovation for your university?

What are some of your key initiatives? What are your key priorities?   From your perspective, what are the key challenges in implementing this vision? How do these fit in with other initiatives on your campus?

(To Listen for: How do they frame the issue? How do they define innovation (competitive advantage, clinical innovations, cutting edge treatments)  How do they conceptualize transformation (creating research centers that advance evidence based treatment etc)

**DEI:**

We know there are AHC /medical schools/cancer centers/are embracing DEI/anti-racism efforts.

Could you talk about what’s happening here? What role, if any, do these efforts play in your overall efforts?

**Community/Patient Engagement/CBPR**

As you know, community and patient engaged research/CBPR is a growing national research initiative with multiple funders mandating that centers implement community engagement offices or cores as part of their research infrastructure.

In your opinion, what are the key barriers to operationalizing equity-based patient and Community Engaged Research in your institution?

What role, if any, do you see community or patient stakeholders’ currently play in overall governance and agenda setting for the AHC?

What institutional mechanisms would you or do you have in place to steer your organizational efforts?

What resources are necessary to successfully facilitate this?

As an administrator with a high-level view of the institution, what are the key challenges to successful implementation of CEnR?

What role do funders play (or not) in successful implementation?

What are some key innovations in your academic health center?

What changes would you like to see happen in the next year to strengthen equity-based patient and community engagement as a sustained committed effort?

**FINAL**

To end the interview, let's shift gears and talk about the internal offices that are most central to implementing CEnR and CBPR at (Insert name). From your perspective, what offices or centers are most involved in these efforts?

How are they implementing these efforts?

In your mind, what makes these offices successful? What are their challenges? What role do individual advocates play in these efforts?

We have covered a lot of material today and we are grateful for your time. As a reminder your name will not be included in the transcript and our intent is to better grasp how leaders like yourself prioritize academic health research, view institutional transformation, and how they are implementing CBPR/CEnR mandates. 

**Governance at the Cancer Center, PRC, and CTSA level**

Autonomy

Integration We are finding that there is a lot of variation in how much autonomy and interaction exists between entities such as Cancer Centers, Prevention Resource Centers, and CTSA’s. From your perspective,

Accountability Primary Stakeholders: who and what entities (accountability processes internally, nationally (how are mandates operationalized within the center), regionally, local level)

Degree of participatory governance, hierarchy, or delegation

Role of Community engagement in center efforts and decision making.

**Governance and power**

Thinking about your health center within the overall institution(university) -how does your center fit within the governance model described?

**Institutional Transformation**

These days there is a lot of emphasis on institutional transformation and innovation in higher education. We want to learn more about your perspective on these efforts

How do you/your institution think about transformation and or innovation?

PROBE: What is your vision? What are your key priorities?

In your view, what are the key factors that 

Internal networks

(For UW we need to understand the broader consortium). Stanford: health center; Morehouse: school of medicine, Stanford: Academic health center; UW: coalitional model that is shifting due to merger at the broader level.

How is it organized?

**External network**

How does your center interact or work with other centers inside your institution?

How does your center interact or work with other centers outside your institution?

**Culture/Narrative**

Within your institution, what are some of the narratives around community engagement that people within your institution speak about?

What are some of the narratives around community engagement told from those outside of your institution?

WE need to add some qualitative assessment of the institutional climate with respect to power relations among faculty

CEO/Executive (Leader)

VP of Research

Organization of the school/mission vision how does research fit in. What does community engagement look like to them?

Try to understand what they demand how it is supplied where authority resides (who do people feel accountable to)

Transparency of policies and procedures. Discretionary practices what do they look like degree of discretion. How do we understand discretion?

Cancer Center director

CTSA Director

Who do people report to? What are the roles they play?

**Questions for CTSA Director, Cancer Directors, PRC Directors and Key Leaders from Each Group**

Tell us a little bit about your Community Engagement Core /PRC/OCOE/CTSA center:

How is it organized?

What is your governance model?

Who are the key players?

What are your funding sources?

How are decisions on budget allocations made?

Think

What power dynamics do you see within the health center?
